# Identification of a Novel TGFβ/PKA Signaling Transduceome in Mediating Control of Cell Survival and Metastasis in Colon Cancer

**DOI:** 10.1371/journal.pone.0019335

**Published:** 2011-05-03

**Authors:** Sanjib Chowdhury, Gillian M. Howell, Ashwani Rajput, Carol A. Teggart, Lisa E. Brattain, Hannah R. Weber, Aparajita Chowdhury, Michael G. Brattain

**Affiliations:** 1 Eppley Cancer Center, University of Nebraska Medical Center, Omaha, Nebraska, United States of America; 2 Department of Surgery, The University of New Mexico, Albuquerque, New Mexico, United States of America; Roswell Park Cancer Institute, United States of America

## Abstract

**Background:**

Understanding drivers for metastasis in human cancer is important for potential development of therapies to treat metastases. The role of loss of TGFβ tumor suppressor activities in the metastatic process is essentially unknown.

**Methodology/Principal Findings:**

Utilizing *in vitro* and *in vivo* techniques, we have shown that loss of TGFβ tumor suppressor signaling is necessary to allow the last step of the metastatic process - colonization of the metastatic site. This work demonstrates for the first time that TGFβ receptor reconstitution leads to decreased metastatic colonization. Moreover, we have identified a novel TGFβ/PKA tumor suppressor pathway that acts directly on a known cell survival mechanism that responds to stress with the survivin/XIAP dependent inhibition of caspases that effect apoptosis. The linkage between the TGFβ/PKA transduceome signaling and control of metastasis through induction of cell death was shown by TGFβ receptor restoration with reactivation of the TGFβ/PKA pathway in receptor deficient metastatic colon cancer cells leading to control of aberrant cell survival.

**Conclusion/Significance:**

This work impacts our understanding of the possible mechanisms that are critical to the growth and maintenance of metastases as well as understanding of a novel TGFβ function as a metastatic suppressor. These results raise the possibility that regeneration of attenuated TGFβ signaling would be an effective target in the treatment of metastasis. Our work indicates the clinical potential for developing anti-metastasis therapy based on inhibition of this very important aberrant cell survival mechanism by the multifaceted TGFβ/PKA transduceome induced pathway. Development of effective treatments for metastatic disease is a pressing need since metastases are the major cause of death in solid tumors.

## Introduction

Colorectal cancer (CRC) is one of the most common malignancies with high incidence rates globally [1] and is the second highest cause of cancer related death among adults in the United States [2]. CRC can be cured by surgery and multimodal treatment in about half of the individuals with this disease (Stages I–III). However, metastasis to distant organs (Stage IV) is the most frequent cause of treatment failure [2]. Recent work has stressed on the importance of the development of inappropriate cell survival signaling for various steps in the metastatic process. Particularly noteworthy in the context of survival signaling in the metastatic process is the importance of aberrant cell survival to successful colonization at metastatic sites in distal organs [3]. Importantly, molecular mechanisms involved in the early stage of metastasis critical for diagnosis and therapy are not well understood [1]. Several key players including the Bcl-2, inhibitor-of-apoptosis (IAP) proteins XIAP and survivin, and the phosphoinositide 3-kinase (PI3K) – AKT/PKB which transmit anti-apoptotic signals in promoting cancer cell growth have been implicated in metastasis [4], [5].

Tumor suppressor genes (TSG) contribute to the induction of apoptosis in response to stress. The failure to induce apoptosis in response to various types of cellular damage has been long recognized as contributing to oncogenesis. One example of TSG loss contributing to cancer formation and progression is TGFβ signaling [2]. The TGFβ signaling pathway has been contributing both negatively and positively in regulating growth inhibition, proliferation, replication, invasion, metastasis, apoptosis, immune surveillance and angiogenesis in a context dependent manner [6]. TGFβ inhibitory/tumor suppressor responses are decreased with increasing progression and in late stage malignancies are often corrupted in a manner that supports invasion and metastasis [6]. While corruption of TGFβ responses to support metastasis implies the presence of functional receptors in these cells, it is equally clear that there are substantial numbers of models ranging from transgenic mice to human cancer xenografts indicating that loss, or attenuation, of receptor expression in a wide variety of tumor types leads to increased malignancy [7], [8], [9], [10]. These results suggest that some subgroups of cancers have pursued a pathway toward malignant progression involving the loss of TGFβ receptor expression while others have, in a yet undetermined fashion, usurped TGFβ signaling to drive malignant progression. There are several examples of TGFβ receptor silencing in clinical samples indicating that TGFβ receptor RI and/or RII (designated TGFβRI and TGFβRII respectively) downregulation are early events in oncogenesis and that loss of receptor expression by epigenetic silencing correlates to malignant progression in subgroups of several types of cancer [11], [12].

We have shown that TGFβ signaling in an early stage non-metastatic colon carcinoma model leads to cell death in colon cancer cells in response to stress in association with inactivation of pAKT and inhibition of the expression of the IAP protein survivin [13]. Complex formation between survivin and another IAP protein XIAP in the cytoplasm in response to stress enables stabilization of XIAP and inhibition of its effector and executioner caspase targets to inhibit cell death [14]. Therefore, given the dominating role of PI3K/AKT signaling in cell survival mechanisms and the association of XIAP and survivin in cancer progression [5], we posited that loss of TGFβ signaling may contribute to enhanced XIAP/survivin expression and consequently loss of TGFβ signaling may be a key to an aberrant survival mechanism permitting metastatic growth at distal organ sites in contrast to the current view that TGFβ TSG is primarily a gatekeeper to prevent oncogenesis.

TGFβ signaling has been shown to activate cAMP- dependent Protein Kinase A (PKA) [15]. PKA plays a dominant role in the integration of multiple signal transduction networks [16] including the ability to disrupt the XIAP/survivin complex through phosphorylation of survivin on Ser^20^ [17].

The molecular mechanisms involved in the TGFβ mediated downregulation of IAP were investigated in order to determine the effects of TGFβ receptor signaling on metastasis in a metastatic orthotopic model of colon carcinoma. We now report the identification of a novel TGFβ/PKA/AKAP mediated transduceome that converges on XIAP function in controlling aberrant cell survival. Additionally, we have shown that TGFβ receptor rescue in highly metastatic cells with epigenetic silencing of TGFβ receptors leads to decreased metastases in a TGFβ/PKA signaling pathway dependent mechanism in a highly metastatic orthotopic colon cancer models in vivo. Rescue of specific tumor suppressor aspects of the TGFβ signaling pathway may provide therapeutic benefits without promoting the cell survival and metastatic effects of TGFβ. If these TGFβ tumor suppressor effects can be mimicked in late stage tumors, therapeutic value against metastatic CRC might ultimately be obtained.

## Results

### TGFβ activates PKA in colon cancer cells

**Figure 1:**
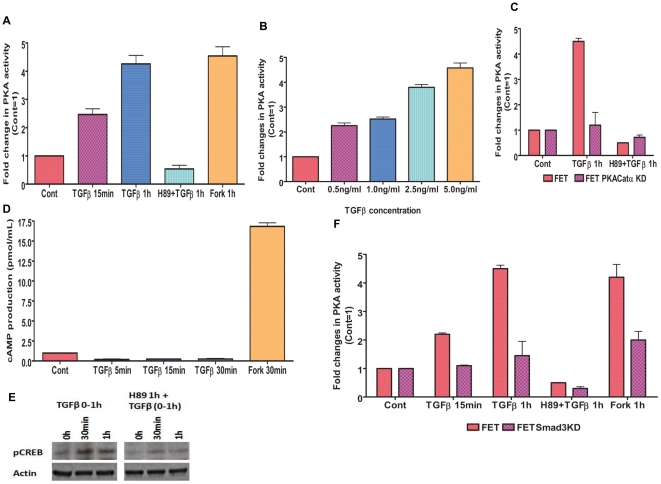
TGFβ activates PKA in colon cancer cells. FET cells treated with TGFβ (5 ng/ml) activated PKA in a time (A) and concentration (B) dependent manner. PKA inhibitor H89 (15 µM) was used to inhibit PKA activation. Forskolin (10 µM) was used as a positive control. Comparison of parental FET and FET PKACatα KD cells to TGFβ mediated PKA activation (C). Knockdown of catalytic α-subunit abrogated PKA activation by TGFβ. PKA activation by TGFβ is independent of cAMP generation as determined by cAMP assay (D). TGFβ leads to CREB activation in FET cells (E). Actin was used as loading control for western blots. PKA activation by TGFβ is Smad3 dependent as determined by knockdown of Smad3 in FET cells (F). The results are expressed as mean ± S.E.M (n = 3). All experiments have been repeated three times independently.

A critical report from the Simeone laboratory noted that TGFβ signaling activates PKA in Mv1Lu cells and mediates control of TGFβ growth inhibitory responses [15]. TGFβ activation of PKA was mediated by a Smad-dependent, cyclic AMP (cAMP)-independent mechanism. This raised the possibility that the TGFβ signaling mediated control of aberrant cell survival observed in the early stage TGFβ-sensitive FET colon carcinoma model might involve the activation of PKA. We hypothesized that TGFβ activates PKA in FET cells in a cAMP independent, Smad dependent mechanism. To this end, FET cells were treated with TGFβ (5 ng/ml) for specified times, and a protein kinase assay was performed for measuring PKA activity (Figure 1A). Following TGFβ treatment, PKA activity increased by approximately 2-fold within 15 min and 4-fold at 1 h. It was observed that TGFβ mediated PKA activation was completely abolished following pretreatment of cells with H89 (15 µM), a pharmacological PKA inhibitor [15]. TGFβ mediated activation of PKA was also concentration dependent (Figure 1B) with maximal activity in FET cells observed at 5 ng/ml TGFβ. To confirm that activation of PKA was downstream of Smad activation by TGFβ, we observed that pretreatment with H89 had no effect on phosphorylation of Smad2 by TGFβ using immunoblot analysis (Figure S1). We developed stable PKA catalytic α subunit shRNA knockdown in FET cells (designated FET PKACatα KD) to validate the H89 response genetically (Figure S2). TGFβ treatment for the specified times was unable to activate PKA in the knockdown cells as opposed to robust activation in the parental FET cells (Figure 1C). Activation of PKA by TGFβ has been shown to be cAMP independent in Mv1Lu cells [15]. Classical PKA activation involves cAMP binding to the regulatory subunits of PKA to trigger dissociation of the catalytic subunits. An alternative mechanism of PKA activation involves the association of IκB with PKA catalytic subunits, thereby maintaining an inactive state of PKA. Upon IκB degradation, there is activation of PKA independent of cAMP activation [18]. We determined whether activation of PKA by TGFβ is dependent upon cAMP activation in FET cells by treating cells with TGFβ and measuring cAMP levels using a non-radioactive cAMP enzyme immunoassay (Figure 1D). It was observed that TGFβ was unable to increase cAMP production in contrast to Forskolin treatment which provided a significant increase in cAMP levels as expected. Next, we examined IκB protein following TGFβ treatment for specified times to determine whether the activation of PKA by TGFβ was due to IκB degradation (Figure S3). The IκB levels remained unchanged following TGFβ treatment. Therefore, PKA is activated by TGFβ in a cAMP and IκB independent manner in FET cells in agreement with the report from Zhang et al (2004). To further confirm the functional significance of TGFβ mediated PKA activation, transcription factor CREB was tested, which has been identified as a direct target of the cAMP-PKA signaling pathway [15]. We observed that TGFβ was able to stimulate the phosphorylation of CREB (Figure 1E) with no change in the total CREB levels (data not shown). Pretreatment of cells with H89 followed by TGFβ exposure significantly reduced the phosphorylation of CREB. Zhang et al (2004) emphasized the role of activated Smad3 in TGFβ mediated PKA activation. We developed stable shRNA Smad3 knockdown in FET cells (designated FET Smad3KD) (Figure S4). TGFβ treatment on these Smad3 knockdown cells was unable to activate PKA (Figure 1F) indicating to the dependence of TGFβ mediated PKA activation on Smad3 as reported earlier [15].

### TGFβ/PKA signaling mediated XIAP downregulation

**Figure 2:**
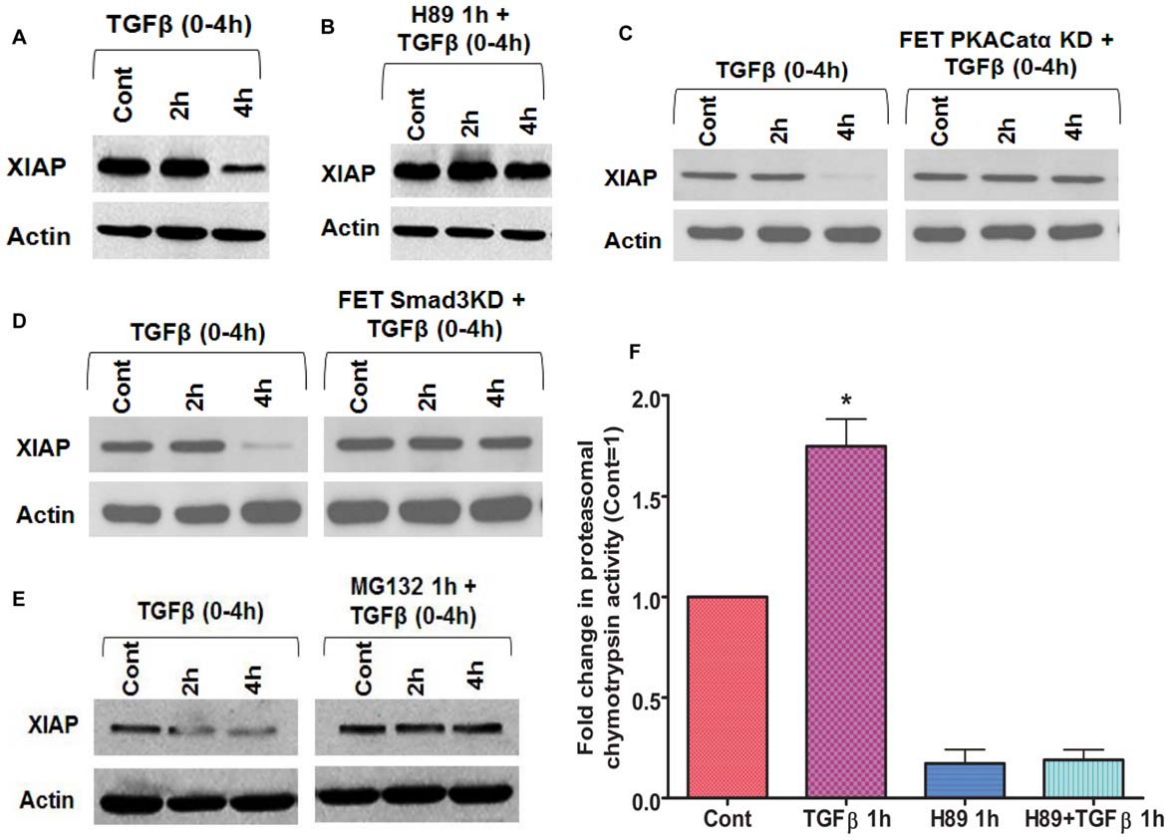
TGFβ/PKA signaling mediated XIAP downregulation. TGFβ (5 ng/ml) treatment for indicated times downregulates XIAP in FET cells (A) and this effect is mediated by PKA as pretreatment with PKA inhibitor H89 prior to TGFβ exposure abrogates XIAP loss (B). Stable shRNA knockdown of PKA catalytic subunit in FET cells leads to abrogation of XIAP loss (C). Stable shRNA knockdown of Smad3 leads to abrogation of XIAP loss in FET cells (D). TGFβ (5 ng/ml) mediated XIAP downregulation is a proteasomal event. Pretreatment with proteasomal inhibitor MG132 (15 µM) abrogated XIAP loss by TGFβ (E). TGFβ mediated activation of PKA leads to activation of chymotrypsin activity within the proteasome and this effect is abrogated by H89 (F). The results are expressed as mean ± S.E.M (n = 3). Actin was used as loading control for western blots. All experiments have been repeated three times independently.

XIAP and survivin are well characterized IAP family members recently documented as metastatic genes [5]. Their concerted role in promoting cell survival and metastasis provides a strong rationale for targeting the expression and/or activity of these IAPs in advanced stages of cancer. An early event associated with PKA activation is phosphorylation of survivin on Ser^20^ in the cytosol, but not in mitochondria [17]. This phosphorylation of survivin has been shown to disrupt the binding interface for XIAP, leading to XIAP degradation. Our data as well as earlier reports indicate that the PKA activation response is an early event following TGFβ treatment and it attains maximal levels within 1 h of TGFβ treatment. We hypothesized that TGFβ causes a disengagement of aberrant cell survival by stimulating PKA mediated disruption of the XIAP/survivin complex. FET cells were treated with TGFβ for the specified times followed by determination of XIAP and survivin (Figure 2A and S5). TGFβ treatment downregulated both XIAP and survivin at the specified times. As survivin phosphorylation has already been shown to be linked to PKA activation, we focused our attention on the regulation of XIAP. Degradation of XIAP was blocked by H89 pretreatment prior to TGFβ exposure for the specified times (Figure 2B). To examine the specificity of the response, we compared the effect of TGFβ on FET PKACatα KD cells and parental FET cells (Figure 2C). TGFβ treatment had no effect on XIAP protein levels in FET PKACatα KD cells indicating the dependence on the PKA catalytic subunit in TGFβ mediated loss of XIAP protein. To confirm the specificity of TGFβ/PKA pathway in this response, FET Smad3 KD cells were treated with TGFβ for the specified times and XIAP protein expression was determined (Figure 2D). Consistent with the Smad3 dependence of TGFβ mediated PKA activation, stable shRNA knockdown of Smad3 abrogated XIAP downregulation. A proteasomal inhibitor (MG132) was used to determine whether loss of XIAP was dependent upon proteasomal degradation (Figure 2E). It was observed that pretreatment of FET cells with MG132 (15 µM) for 1 h prior to TGFβ treatment completely abolished the XIAP degradation indicating that XIAP is degraded within the proteasome. PKA is an important regulator of proteasomal activity and PKA has been shown to co-purify with the proteasome [19], [20]. We hypothesized that TGFβ/PKA mediated regulation of proteasomal activity may provide another layer of control of IAP expression (beyond the PKA induced phosphorylation of survivin on Ser^20^). Earlier reports indicate that PKA phosphorylates the Rpt6 ATPase, which unfolds and transports substrates into the proteasome and this phosphorylation enhances the chymotrypsin proteasomal activities of the proteasome [21]. To this end, we determined whether TGFβ mediated PKA activation is able to increase the chymotrypsin-like proteasomal activity using a proteasomal activity assay. TGFβ treatment of FET cells selectively stimulated the chymotrypsin-like activity of the proteasome within 1 h (Figure 2F). H89 pretreatment prior to TGFβ exposure completely abrogated the basal level of the proteasomal chymotrypsin-like activity and completely abolished the stimulatory effects of TGFβ on the proteasome.

Collectively, these data lead to the hypothesis that the TGFβ/PKA signaling regulates XIAP expression at multiple levels with activation of PKA leading to phosphorylation of key proteasomal components that promote proteasomal degradation of XIAP.

### AKAP regulates TGFβ/PKA mediated XIAP downregulation

**Figure 3:**
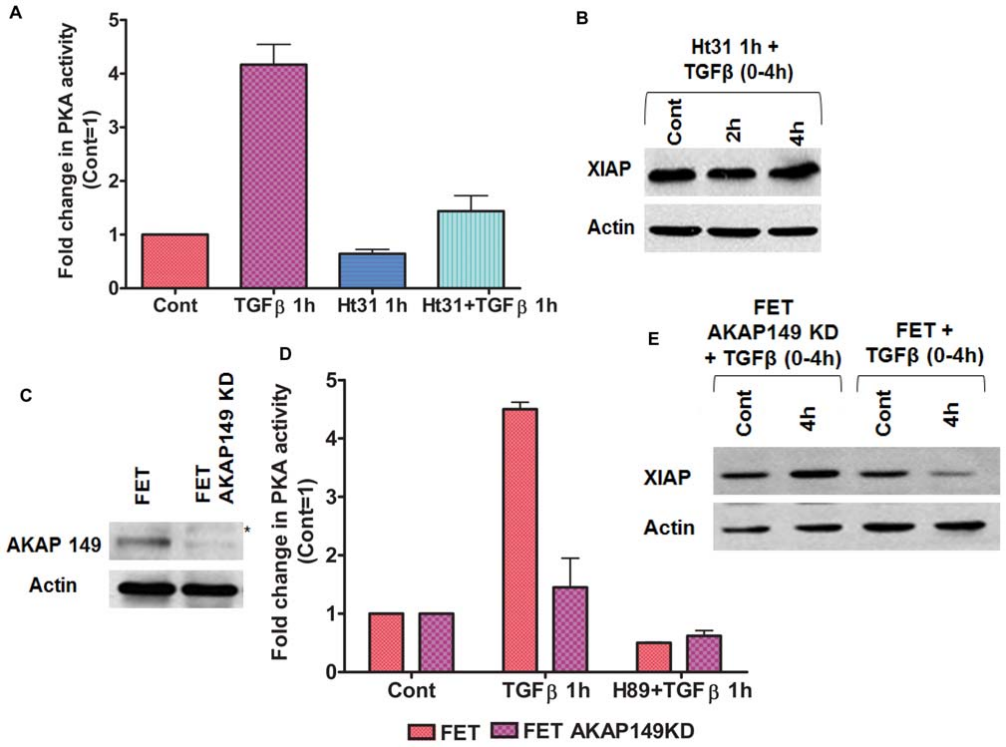
AKAP regulates TGFβ/PKA mediated XIAP downregulation. AKAP inhibitor Ht31 (25 µM) treatment for 1 h prior to TGFβ exposure abrogates TGFβ mediated PKA activity (A) and TGFβ mediated XIAP loss (B). AKAP149 siRNA knockdown was done on FET cells (C). Knockdown of AKAP149 leads to abrogation of TGFβ mediated PKA activity (D) and XIAP downregulation (E). The results are expressed as mean ± S.E.M (n = 3). Actin was used as loading control for western blots. All experiments have been repeated three times independently.

There is an abundance of A-kinase anchoring proteins (AKAPs) which compartmentalize the PKA and other enzymes to the vicinity of specific cellular organelles for carrying out enzyme function. We hypothesized that AKAPs are a critical component in TGFβ/PKA mediated XIAP downregulation. It is well documented that the subcellular localization of PKA regulatory subunits (PKARI and/or PKARII) is mediated through interactions with AKAPs [22]. AKAP inhibitor Ht31, a synthetic thyroid anchoring peptide has been shown to be a very potent competitive inhibitor of PKARII/AKAP interaction, thereby preventing PKA anchoring [23]. To ensure that the Ht31 was specifically targeting AKAP-PKA interactions, we performed PKA activity assays on FET cells to determine the extent of PKA activation following pretreatment with Ht31 (25 µM) prior to TGFβ exposure (Figure 3A). In agreement with earlier studies [15], Ht31 was able to block the TGFβ mediated PKA activation. Since AKAP-PKA interaction appears to be a requirement for TGFβ mediated PKA activation, we reasoned that these interactions might also be required for downstream signaling events leading to XIAP loss (Figure 3B). Addition of Ht31inhibitor for 1 h prior to TGFβ treatment for specified times completely abrogated the XIAP loss.

Since the AKAP family includes more than 50 members [24], the challenge was to identify an individual AKAP that was responding to the TGFβ/PKA effects on XIAP. After initial screening for the presence of different AKAP family members in FET cells, we found AKAP149 protein expression in these colon cancer cells. AKAP149 (also termed D-AKAP1 and AKAP121 in mouse) has been identified as a membrane protein of the mitochondria [25]. It has been reported that mitochondrial AKAP121 is involved in targeting cAMP activated PKA to the outer mitochondrial membrane (OMM) and plays a role in mitochondrial biogenesis and survival [26]. We hypothesized based on the understanding that the mitochondrial XIAP and survivin move to the cytoplasm following a stress response that AKAP149 might be regulating TGFβ/PKA mediated XIAP degradation. Expression of an AKAP149 small interfering RNA (siRNA) prevented the TGFβ mediated PKA activation (Figure 3C–D). Further, we observed that TGFβ was unable to downregulate XIAP protein in AKAP149 siRNA knockdown cells compared to parental FET control cells (Figure 3E). This pro-apoptotic function of AKAP149 is in contrast to the known function of mitochondrial AKAP121 (or its human homologue AKAP149) in promoting survival.

We next questioned whether the effect of AKAP149 was selective in this TGFβ/PKA effect. While AKAP-PKA interaction is a global phenomenon, validation of AKAP149 as selectively regulating TGFβ/PKA signaling would be a novel extension of our understanding of the TGFβ/PKA mediated XIAP downregulation. AKAP220 has been shown to regulate protein phosphatase 1 (PP1) activity by coordinating the location of PKA and PP1 catalytic subunit [27]. We silenced the expression of AKAP220 in FET cells with AKAP220 siRNA. These transfected cells when treated with TGFβ had no effect on either the induction of PKA activity or XIAP downregulation (data not shown). Taken together, AKAP scaffolding plays a pivotal role in the TGFβ/PKA pathway mediated XIAP downregulation with mitochondrial localized AKAP149 being necessary for the TGFβ/PKA mediated response.

### Involvement of PP2A in TGFβ/PKA mediated XIAP downregulation

**Figure 4:**
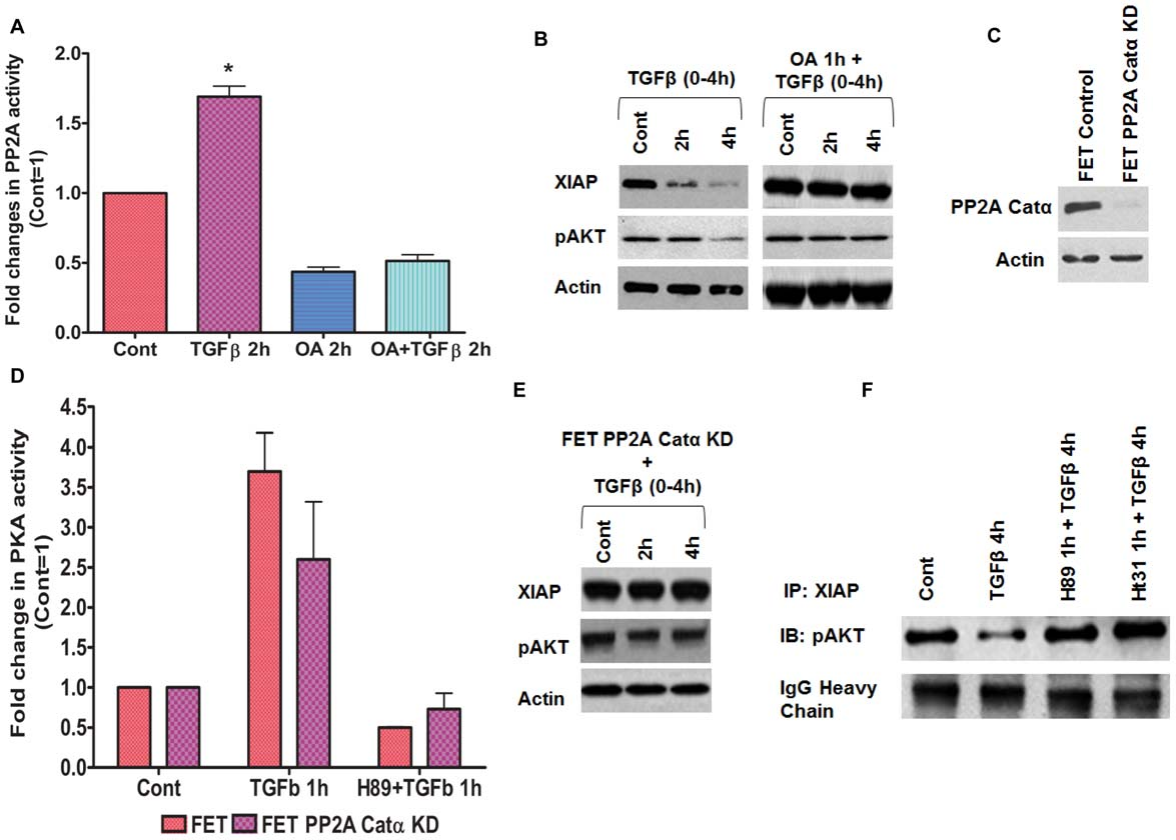
Involvement of PP2A in TGFβ/PKA mediated XIAP downregulation. FET cells were treated with TGFβ (5 ng/ml) for the indicated time and PP2A activity was determined using PP2A activity assay protocol as described in materials and methods (A). PP2A inhibitor okadaic acid (OA, 50 nM) was used to inhibit PP2A. Pretreatment with OA prior to TGFβ (5 ng/ml) treatment abrogated TGFβ mediated XIAP loss and dephosphorylation of AKT (B). Stable shRNA knockdown of PP2A catalytic α-subunit were done on FET cells (C). Knockdown of PP2A catalytic α-subunit led to abrogation of TGFβ (5 ng/ml) mediated XIAP downregulation without affecting PKA activity (D–E). XIAP immunoprecipitation studies in FET cells treated with TGFβ (5 ng/ml) or pretreated with H89 or Ht31 for 1 h prior to TGFβ exposure. XIAP dissociates from pAKT following TGFβ treatment for indicated time (F). Inhibiting the PKA activity by H89 or AKAP-PKA interaction with Ht31 abrogates the TGFβ mediated XIAP-pAKT dissociation.

It has been observed that activation of PKA leads to the inactivation of AKT through dephosphorylation by protein phosphatase 2A (PP2A) [28]. This led to the hypothesis that TGFβ mediated activation of PKA is responsible for the repression of AKT activation that we previously reported as a consequence of TGFβ signaling [13]. Consequently, we determined PP2A activity in FET cells (Figure 4A). TGFβ treatment for specified times significantly increased the PP2A activity. PP2A selective inhibitor okadaic acid (OA, 50 nM) was able to completely abolish the PP2A activation when treated alone or pretreated prior to TGFβ exposure. Next, we tested the role of TGFβ in mediating AKT inactivation and XIAP loss (Figure 4B). It was observed that TGFβ mediated dephosphorylation of AKT is PP2A dependent as reflected by the ability of OA to block the TGFβ mediated dephosphorylation of AKT. XIAP downregulation was also abolished by OA treatment. Although PP2A is a tumor suppressor involved in the control of several cell survival pathways [29], documentation of a role in subverting cell survival signaling within the TGFβ pathway is a novel extension of PP2A function.

To further validate the role of PP2A in TGFβ/PKA signaling, we generated stable shRNA PP2A catalytic subunit knockdown in FET cells designated FET PP2A Cat KD (Figure 4C). TGFβ treatment of FET PP2A Cat KD cells showed PKA activation indicating that PKA activation is upstream of PP2A activation (Figure 4D). However, stable shRNA knockdown of the PP2A catalytic subunit completely abolished TGFβ mediated XIAP inhibition and showed sustained phosphorylation of AKT as well (Figure 4E). FET PKACatα KD cells treated with TGFβ also showed a sustained phosphorylation of AKT (data not shown).

While inhibition of AKT activation would have potential for many effects on cell survival, one effect that is of particular interest as related to TGFβ/PKA function is in the context that it targets XIAP stability. A previous study reported that XIAP is phosphorylated by AKT on Ser^87^ which leads to increased stabilization of XIAP and decreased apoptosis of ovarian cancer cells in response to cisplatin [30]. We reasoned that PP2A inhibition of AKT activity may represent an additional mechanism for the disruption of the stabilization of XIAP that is targeted by the TGFβ/PKA pathway through enhancement of PP2A activity. To this end, FET cells were treated with TGFβ or pretreated with different TGFβ/PKA pathway inhibitors prior to TGFβ exposure for specified times and immunoprecipitated for XIAP and immunoblotted for possible proteins bound to XIAP. We found an association of XIAP with pAKT (Figure 4F). Following TGFβ treatment, there was a significant dissociation of the XIAP-pAKT complex. However, pretreatment with H89 or Ht31 prior to TGFβ exposure completely abrogated XIAP-pAKT dissociation of the two proteins indicating that TGFβ/PKA signaling mediated inactivation of AKT destabilizes XIAP leading to its proteasomal degradation. Therefore, using inhibitor and stable knockdown studies, we underscore the novel finding that TGFβ/PKA signaling mediates loss of XIAP protein by a PKA-PP2A dependent repression of AKT activation leading to XIAP loss.

### TGFβ receptor reconstitution leads to decreased metastatic colonization: Impact on TGFβ/PKA signaling

**Figure 5:**
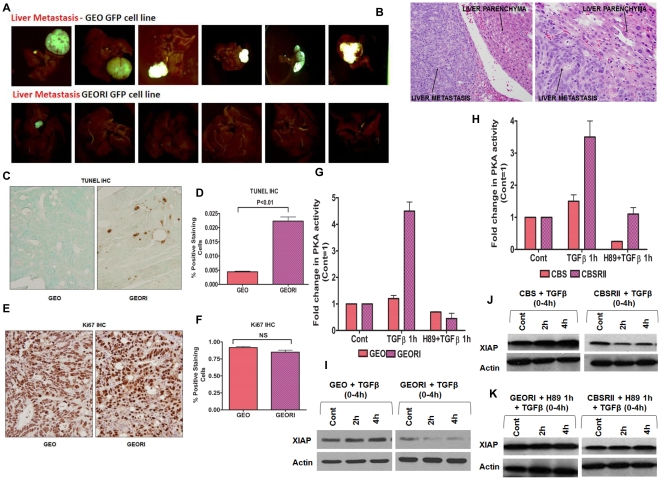
TGFβ receptor reconstitution leads to decreased metastatic colonization: Impact on TGFβ/PKA signaling. Comparison of GFP images of metastatic progression into liver in GEO and GEORI mice (A). GEO Colon Cancer Liver H&E staining showing liver metastasis (B). Comparison of primary tumor sections of GEO and GEORI mice by TUNEL assay staining as mentioned in Materials and Methods to determine their apoptotic rates (C). The apoptotic rates were quantified (D). Comparison of primary tumor sections of GEO and GEORI mice by Ki-67 staining as mentioned in Materials and Methods to determine their rates of proliferation (E). The proliferation rates were quantified (F). TGFβ receptor reconstitution leads to a change in PKA activity. Comparison of PKA activity in highly metastatic GEO cells vs. poorly metastatic GEORI cells (G) and in highly metastatic CBS cells vs. poorly metastatic CBSRII cells (H). Comparison of TGFβ (5 ng/ml) exposure between GEO and GEORI cells (I) and CBS and CBSRII cells (J). PKA inhibitor H89 abrogated TGFβ mediated XIAP loss in GEORI and CBSRII cells (K).

We have developed technology for orthotopic colonic implantation of human colon cancer lines in athymic mice that allows for reproducible quantitative analysis of metastasis to the liver and lungs [31], [32], [33]. The metastatic pattern displayed in this model system reflects the nature of metastatic spread in human patients. Highly metastatic GEO colon cancer cells were used to further understand the impact of TGFβ/PKA signaling on metastasis. GEO cells have attenuated TGFβRI expression and thus attenuated TGFβ tumor suppressor signaling as well [8]. We have utilized colonic orthotopic implantation of subcutaneously grown xenografts to characterize factors that influence the extent of metastasis to liver and lungs without affecting invasion at the primary tumor site [31], [33]. GEO cells were found to be highly metastatic in this orthotopic model as reflected by metastatic colonization in 53% of the implanted animals (Table 1). Rescue of receptor attenuation in GEO cells by transfection of a TGFβRI expression vector (designated GEORI) resulted in the reduction of metastatic incidence to about 20% of animals implanted without affecting invasion at the primary site as both GEO and GEORI transfected animals gave rise to an invasive primary tumor in 100% of implanted animals. GEO transplanted animals developed robust liver metastasis compared to GEORI (Figure 5A). Hematoxylin and Eosin (H&E) staining showing liver metastasis in GEO cells is shown in Figure 5B. The characterization of GEO tumors showed increased cell survival signaling as reflected by lower TUNEL rates relative to GEORI tumors indicating a repression of metastatic colonization by TGFβ tumor suppressor signaling is associated with repression of cell survival signaling in vivo (Figure 5C–D). Further, no change in Ki67 IHC staining was observed between highly metastatic GEO and poorly metastatic GEORI primary tumors indicating no difference in proliferation rate in vivo between GEO and GEORI tumors (Fig 5E–F). The in vivo findings indicate that the restoration of TGFβ receptor suppresses metastatic competence in GEO cells at the level of metastatic colonization as opposed to preventing invasion.

**Table 1:** Results of orthotopic implantation demonstrating the primary invasion and metastases based on histologic evaluation.

Cell Line	Primary	Liver Mets	Lung Mets	Liver or Lung Mets
GEO	38/38 (100%)	17/38 (45%)	8/38 (21%)	20/38 (53%)
GEOR1	30/30 (100%)	6/30 (20%)	0/30 (0%)	6/30 (20%)

A key to capitalizing on the repression of metastasis by TGFβ signaling is the elucidation of the molecular mechanism by which metastasis is inhibited. Based on the decrease in metastatic incidence with TGFβ receptor reconstitution leading to rescue of TGFβ signaling, we hypothesized that TGFβ/PKA mediated XIAP downregulation requires an intact TGFβ signaling mechanism which is lost in the highly metastatic GEO cells. However, rescue of TGFβ signaling by receptor reconstitution in GEORI cells leading to decreased metastatic incidence should increase TGFβ/PKA signaling and its downstream effects on XIAP. GEO cells with attenuated TGFβ signaling had no PKA activation with TGFβ exposure (Figure 5G). However, GEORI cells with functional TGFβ signaling due to receptor reconstitution had a robust increase in PKA activation by TGFβ which was about 4-fold higher than the control. A second colon carcinoma cell line (CBS cells) with attenuated TGFβRII signaling [34] also showed reduced metastasis after rescue of receptor deficiency (data not shown). A similar response in PKA activity was observed in highly metastatic CBS cells compared to the poorly metastatic CBSRII cells after TGFβ treatment in vitro (Figure 5H).

Following this observation, we hypothesized that the highly metastatic GEO and CBS cells would be resistant to the TGFβ/PKA signaling mediated loss of XIAP protein. We reasoned that the inability of TGFβ signaling to induce PKA activation in these cells due to receptor inactivation would also prevent its downstream effects on XIAP thereby making these cells more metastatic through increased pro-survival signaling. While GEO and CBS both showed no response to TGFβ treatment at specified times (Figure 5I–J), GEORI and CBSRII cells showed XIAP downregulation for similar treatments (Figure 5I–J). To validate the role of PKA activation in XIAP downregulation by TGFβ, we used H89 pretreatment prior to TGFβ exposure in GEORI and CBSRII cells (Figure 5K). In line with our observations in FET cells, both GEORI and CBSRII cells pretreated with H89 showed abrogation of TGFβ/PKA mediated XIAP downregulation. Thus, TGFβ receptor restoration in deficient cells was able to reactivate the TGFβ/PKA signaling pathway in poorly metastatic cells leading to XIAP loss. These cells mimicked the observation from FET cells, which has a functional TGFβ/PKA signaling. Therefore, the in vitro results are consistent with the in vivo observation that TGFβ receptor rescue reactivates the attenuated TGFβ signaling in controlling cell survival and metastasis. Taken together, these findings along with clinical evidence of TGFβ receptor silencing suggests that reconstitution of TGFβ receptor expression could lead to inhibition of growth and/or induce apoptosis in highly progressed metastatic colon cancer cells by the TGFβ/PKA signaling pathway.

### TGFβ/PKA signaling controls cell survival

**Figure 6:**
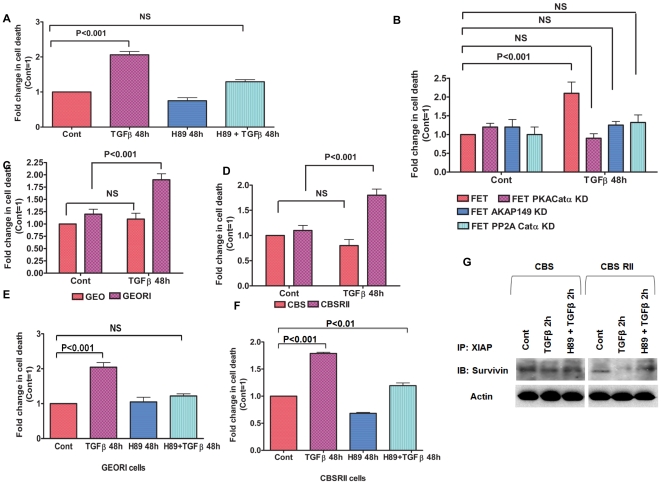
TGFβ/PKA signaling controls cell survival. TGFβ induces cell death in FET cells mediated by the PKA as determined by DNA fragmentation assay (A). Pretreatment with PKA inhibitor H89 (1 µM) for 1 h prior to addition of TGFβ (5 ng/ml) in the same media for indicated time abrogated the TGFβ mediated cell death. Comparison of cell death by DNA fragmentation assays between parental FET and FET PKACatα, FET AKAP149 and FET PP2A Catα KD cells in response to TGFβ (5 ng/ml) exposure for indicated time (B). Differential levels of cell death induced by TGFβ (5 ng/ml) exposure for indicated time in between GEO/GEORI cells (C) and CBS/CBSRII cells (D). TGFβ induces cell death in GEORI (E) and CBSRII (F) cells mediated by the PKA as determined by DNA fragmentation assay. Pretreatment with PKA inhibitor H89 (1 µM) for 1 h prior to addition of TGFβ (5 ng/ml) in the same media for indicated time abrogated the TGFβ mediated cell death. TGFβ mediates dissociation of XIAP-survivin complex in poorly metastatic CBSRII cells compared to highly metastatic CBS cells in a PKA dependent manner (G).

Recently, we showed that TGFβ signaling leads to cell death in FET cells in vitro in response to stress [13]. Based on this background and the current findings regarding AKAP-PKA transduceome signaling in XIAP downregulation, we hypothesized that the tumor suppressor effects of TGFβ can be mediated through the TGFβ/PKA signaling axis. Using FET cells as an established model, we tested the effects of TGFβ treatment on cell death to further understand the role of the TGFβ/PKA signaling pathway in abrogating cell survival. TGFβ treatment for 48 h showed approximately 2-fold increase in cell death (Figure 6A). To better understand the impact of PKA in the TGFβ effect, cells were pretreated with low dose of H89 (1 µM) prior to TGFβ treatment. Compared to control FET cells, inhibiting the PKA activation with H89 significantly decreased the cell death in these cells. To further confirm the role of PKA, FET PKACatα KD cells were treated with TGFβ for the specified times and cell death was assessed in comparison to FET cells (Figure 6B). PKA catalytic subunit knockdown completely abrogated the cell death by TGFβ treatment indicating that PKA activation is critical in TGFβ mediated cell death.

Since AKAP149 was observed to be a critical player in the TGFβ/PKA mediated XIAP loss, we reasoned that AKAP149 might also be critical for the TGFβ/PKA mediated cell death. Silencing of AKAP149 expression using AKAP149 siRNA in FET cells followed by TGFβ treatment to assess cell death was performed (Figure 6B). AKAP149 knockdown completely abrogated cell death in these cells. The siRNA knockdown studies indicate that AKAP149 is selective for TGFβ/PKA signaling in FET cells.

Since TGFβ mediated PKA activation leads to activation of PP2A followed by AKT dephosphorylation and XIAP downregulation, we reasoned that inhibiting PP2A should also alter the cell death response to TGFβ. For this reason, FET PP2A Cat KD cells were compared with parental FET cells for effects on cell death by TGFβ signaling (Figure 6B). Indeed, stable knockdown of PP2A catalytic subunit completely abrogated the PP2A mediated cell death.

GEO cells reconstituted with TGFβRI showed a decrease in metastatic capability. Consequently, we determined whether receptor reconstitution also causes an increase in functional TGFβ tumor suppressor signaling leading to increased cell death in these cells. Comparison of GEO and GEORI cells with respect to cell death by TGFβ treatment for 48 h was performed (Figure 6C) and similarly, comparisons were performed with CBS and CBSRII cells as well (Figure 6D). Death assays revealed a striking difference in their responsiveness to TGFβ induced cell death. While the GEO and CBS cells were completely resistant to TGFβ mediated cell death; the GEORI and CBSRII cells showed significant increases in cell death following TGFβ treatment. We hypothesized that the increase in cell death due to receptor reconstitution was due to the restoration of TGFβ/PKA signaling. To this end, GEORI and CBSRII cells were treated with TGFβ or pretreated with H89 prior to TGFβ exposure and cell death was assessed (Figure 6E–F). In both GEORI and CBSRII cells, H89 was able to abrogate the TGFβ response indicating the importance of TGFβ/PKA signaling in controlling cell survival in these cells.

We hypothesized that the TGFβ/PKA mediated XIAP downregulation would require dissociation of the XIAP and survivin complex prior to XIAP degradation. We compared the endogenous levels of the XIAP/survivin complex in CBS and CBSRII cells and determined whether TGFβ was able to dissociate the XIAP/survivin complex in a PKA dependent manner (Figure 6G). CBS cells (TGFβ signaling deficient) had higher levels of survivin bound to XIAP compared to CBSRII cells with restored TGFβ signaling. Treatment of TGFβ completely dissociated survivin from XIAP in CBSRII cells while survivin remained bound to XIAP in CBS cells. Blocking PKA activation by H89 significantly abrogated the TGFβ mediated XIAP/survivin complex dissociation in accordance with the well documented role of PKA in dissociation of the XIAP/survivin complex [17]. Most XIAP/survivin complex studies have been performed using overexpression approaches as only a small fraction of the total cellular XIAP and survivin form a complex. We used the endogenous protein as opposed to the other approach since overexpression might lead to artifacts in the interaction. Therefore, TGFβ was able to dissociate XIAP/survivin complex through the TGFβ/PKA signaling in poorly metastatic cells which have a restored TGFβ signaling as opposed to their highly metastatic counterparts deficient in functional TGFβ signaling.

## Discussion

### TGFβ signaling and aberrant cell survival

**Figure 7:**
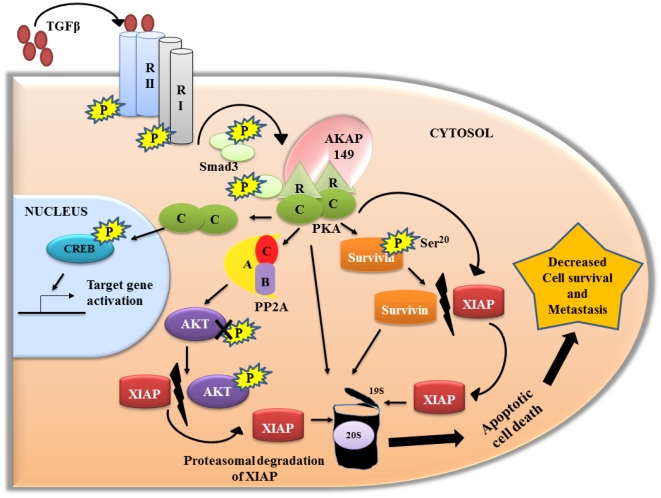
Schematic representation of TGFβ/PKA transduceome signaling.

We have identified a novel mechanism of TGFβ tumor suppressor signaling pathway that is capable of counteracting aberrant cell survival. This TGFβ/PKA/AKAP149 dependent transduceome involves a TGFβ initiated series of kinase and phosphatase events that sequentially converge on the inhibition of XIAP function in promoting cell survival and thereby permitting cell death in response to stress. The proposed mechanism involves the activation of PKA in a manner that is independent of cAMP, but AKAP and Smad3 dependent. PKA activation results in PP2A mediated dephosphorylation of pAKT followed by destabilization of XIAP resulting in its proteasomal degradation (Figure 7). The ability of XIAP to directly inhibit caspases in vivo [35] makes it a critical element in the control of apoptotic threshold in cancer cells. More recently, PKA was found to mediate compartmentalized regulation of survivin on Ser^20^ selectively in the cytosol but not in mitochondria leading to the control of survival signals through degradation of the IAPs [17]. A critical aspect of this novel TGFβ/PKA pathway is the implication that the TGFβ mediated activation of an AKAP/PKA complex initiates a multi-functional cascade directed at disruption of XIAP mediated cell survival involving several different targets including PP2A and the proteasome along with cytosolic survivin.

The tetrameric PKA holoenzyme is a Ser/Thr kinase consisting of two catalytic (C) subunits bound to two (R) subunits (RIα/β and RIIα/β) that renders it inactive [16]. Classically, following elevation of cAMP, the C-subunits disengage from the R-subunits and actively phosphorylate proteins in the cellular vicinity [27]. The correct sub-cellular localization of the PKA holoenzyme within cellular compartments is a function of scaffolding and anchoring proteins AKAPs [27] which contribute to the spatio-temporal regulation of second messenger signaling events [24]. Similar to Zhang et al (2004), we found that AKAP-PKA interaction was required for PKA activation by TGFβ. Earlier work has suggested that AKAPs are required for subcellular localization of PKA and interaction with Smads [15]. We identified a specific mitochondrial localized AKAP149, as having a critical role in TGFβ mediated PKA activation. Knockdown of AKAP149 abrogated TGFβ induced PKA activation and downstream signaling leading to XIAP loss. We demonstrated for the first time that AKAP149/PKA was directly involved in the TGFβ's ability to induce cell death in colon cancer cells. Our study demonstrates that AKAP149 might have context dependent effects based on the mechanism of PKA activation. The cAMP mediated AKAP121/PKA signaling to mitochondria inhibits apoptosis [26]. However, we have demonstrated here that AKAP149 is also involved in a pro-apoptotic role during TGFβ mediated PKA activation independent of cAMP as evidenced by AKAP149 siRNA studies.

An intriguing observation made in our studies which originally showed that TGFβ signaling inhibits survivin expression was that TGFβ signaling also mediated inactivation of AKT [13]. The activation of PKA has been shown to inactivate AKT through dephosphorylation by PP2A [28]. Consequently we tested the hypothesis that inactivation of AKT is dependent upon PP2A in our studies of TGFβ/PKA mediated control of XIAP expression. PP2A is ubiquitously expressed as a trimeric complex composed by a C-catalytic subunit, an A-scaffolding subunit and a B-targeting subunit which is drawn from among several families of multiple isoforms [36] . PP2A has been observed to have a tumor suppressor function [29] playing a critical role in apoptotic cell death through differential interactions with Bcl2 and caspase3. TGFβ has been shown to induce G1 arrest mediated through PP2A [37]. Studies with the PP2A selective inhibitor okadaic acid (OA) have shown that it acts as a tumor promoter in mouse skin carcinoma [38]. We demonstrated that TGFβ mediated activation of PKA was upstream of PP2A activation by stable transfection of FET cells with PP2A catalytic shRNA or treatment with OA resulting in PP2A mediated XIAP degradation through inactivation of AKT. Although PP2A is a tumor suppressor involved in the control of several cell survival pathways, documentation of a role in subverting cell survival signaling within TGFβ function is a novel extension of its function.

We have shown that XIAP is associated with pAKT and this association is disrupted following TGFβ treatment (Figure 4F). AKT along with some of its substrates constitute a major cell survival pathway [30]. Importantly, the anti-apoptotic functions of AKT have been proposed to be at the post-mitochondrial level. It has been shown to directly phosphorylate and inactivate caspase 9 and Bax [30]. XIAP inhibits death-signaling pathways also at the post-mitochondrial level [30] and has been documented as a physiological substrate of AKT. In HEK293 cells, AKT physically associates with XIAP and phosphorylates XIAP on Ser^87^, thereby stabilizing XIAP and preventing it from auto-ubiquitination [30]. We have now demonstrated that pAKT and XIAP are in a complex and TGFβ is able to modulate the complex formation. Further, we observe that dissociation of complex formation by TGFβ was inhibited by H89 and Ht31. This strongly supports the notion that TGFβ exerts its effects on cell survival through the TGFβ/PKA transduceome signaling.

In association with direct regulation of XIAP by TGFβ/PKA signaling, we also demonstrated that chymotrypsin-like activity of the proteasome is induced by TGFβ in a PKA dependent manner. XIAP can be auto-ubiquitinated and degraded when treated with DNA damaging agents like chemotherapeutic drugs [30]. The fact that XIAP has an E3-ligase which could act as a scaffold for EI or E2 ubiquitination enzymes is consistent with XIAP as a potential proteasomal target. PKA is an important kinase in proteasome phosphorylation and the regulation of metabolic function [20]. Collectively, these data lead to the indicate that the TGFβ/PKA transduceome regulates XIAP and survivin expression at multiple levels with activation of PKA complex leading to phosphorylation of key proteasomal components such as Rpt6 [21] which might contribute to the ubiquitin-proteasome mediated degradation of XIAP as well as other related cell survival mediators.

### TGFβ receptor reconstitution suppresses metastasis

We have made the novel observation that reconstitution of TGFβ receptor TGFβRI in highly metastatic GEO colon cancer cells rescues TGFβ signaling and inhibited metastatic colonization from orthotopic xenografts. This observation raises the therapeutic possibility that TGFβ can suppress metastatic competence after this advanced state of progression is attained in cells with attenuated type I and/or II TGFβ receptor expression (designated TGFβRI or TGFβRII).

We have made the novel observation that rescue of attenuated receptor expression does inhibit metastatic capability in GEORI (reconstituted with RI) transplanted orthotopic models in vivo even though invasion at the primary tumor site was maintained (Table 1). This finding indicates that the effect of receptor reconstitution was on the ability to form progressively growing colonies at the distal site rather than preventing escape from the primary tumor. This result also suggested that the inhibition of distant organ colonization might be related to stress induced cell death associated with regenerated TGFβ signaling in response to the foreign micro-environment for growth of the colon cancer cells in the liver and lungs. These results are significant because they raise the possibility that regeneration of attenuated TGFβ signaling leading to activation of the TGFβ/PKA transduceome would be an effective strategy to target the treatment of metastasis. As discussed in the introduction, epigenetic loss of TGFβ receptors is a frequent occurrence in a broad array of different types of cancer. Thus, there is the potential for translating our novel observations into the clinic. To this end, we found that pancreatic, breast and colon cancer cell lines frequently exhibited loss of TGFβRII and/or RII due to transcriptional repression. We identified HDAC inhibition as mechanism for rescue of TGFβ receptor expression in these histological types of cancer [39], [40], [41], [42], [43], [44].

Studies on different cancer types provide extensive support for loss of TGFβ receptor expression as an important contributor to tumor progression in subgroups of several types of cancer [11], [12]. However, there is also evidence from additional clinical studies indicating that other subgroups exist within the same histopathological types of cancer that utilize corrupted TGFβ signaling via TGFβRI and TGFβRII as a means of contributing to cancer progression [45]. Thus, various subgroups of breast and colon cancer have evolved different strategies for generating malignant progression. The existence of corruption of TGFβ signaling as a mechanism for malignant progression does not preclude the clinical importance of loss of TGFβ tumor suppressor signaling due to deficiency of receptor expression as a potential target for cancer therapy. In fact, the evidence for the importance of receptor loss in clinical cancer is, as indicated above, significantly more developed than the evidence for corrupted TGFβ signaling which is largely restricted to in vitro investigations at this point.

In an effort to determine the relationship between the TGFβ/PKA transduceome and the capability of a tumor for metastatic spread, we investigated the molecular aspects of the TGFβ/PKA transduceome signaling in the context of metastatic progression in highly metastatic GEO and CBS cells and their poorly metastatic counterpart GEORI and CBSRII where receptor reconstitution has led to a decrease in metastatic spread. Since metastatic deposits, like primary cancers, are faced with hypoxic stress and must face the additional challenge of growth in a foreign microenvironment, it seems likely that even established metastases must retain their aberrant survival characteristics.

Striking difference in TGFβ/PKA signaling activity and downstream signaling leading to XIAP downregulation was observed when highly metastatic (GEO, CBS) and their poorly metastatic colon cancer counterparts (GEORI and CBSRII) were compared. Therefore, reconstitution of TGFβ receptors in metastatic models with attenuated TGFβ signaling leading to metastasis might also reconstitute TGFβ/PKA signaling in established metastases which could affect their survival. These lines of evidence lead to the consideration that the TGFβ/PKA transduceome is a critical component in steady-state suppression of abnormal survival signaling that prevents formation of metastases by disrupting the XIAP/survivin cell survival pathway.

In conclusion, understanding drivers for metastasis in human cancer is important for potential development of therapies to treat metastases (most prominent cause of death from solid cancers). The role of loss of tumor suppressor activities in the metastatic process is essentially unknown. Presently, loss of TGFβ signaling is largely regarded as a driver for transition from a benign to a malignant state subsequently leading to metastatic spread by other mechanisms or even by usurping aspects of TGFβ signaling for oncogenic mechanisms such as the acquisition of EMT. However, we have now shown that loss of TGFβ tumor suppressor signaling is a driver for metastatic colonization of distant organs from the primary tumor. Importantly, loss of TGFβ signaling does not affect invasion as reflected by retention of a 100% rate of invasion at the primary site of metastatic cells in which TGFβ signaling has been rescued and metastatic potential inhibited. This indicates that while loss of TGFβ signaling enables the transition from a benign to a malignant state its loss does not drive the earliest step of the metastatic process.

However, we have shown here that loss of TGFβ signaling is necessary to allow the last step of the metastatic process- colonization of the metastatic site. Moreover, we have identified a novel TGFβ tumor suppressor pathway that acts directly on a known cell survival mechanism that responds to stress with the survivin/XIAP dependent inhibition of caspases that effect apoptosis. This survival mechanism is apparently not necessary for malignant behavior at the primary site once the cancer cells have made the transition from the benign state since its inhibition by restoration of TGFβ tumor suppressor signaling does not alter malignant behavior at the primary site.

Our work indicates the potential for developing anti-metastasis therapy based on inhibition of this very important aberrant cell survival mechanism by this novel, multifaceted TGFβ induced pathway. Our previous body of work showing that HDACi's act at least in part through the rescue of TGFβ receptor I and II expression and TGFβ tumor suppressor activity in a wide variety of cancer cells indicates the clinical potential of this concept.

Since TGFβ signaling responses can be supportive of events that support malignant progression such as EMT and suppression of TGFβ signaling as a therapy for cancer has been supported by experimental evidence, therapeutic strategies for the inhibition of TGFβ signaling in cancer therapy are currently being pursued. However, our work indicates that the relationship between TGFβ signaling and tumor suppressor function may be governed by context even in the later stages of cancer. The concept that the disruption of the balance between TGFβ tumor suppressor activity and the survivin/XIAP cell stress response enables metastatic colonization of distant organs raises the concern that therapies aimed at inhibition of TGFβ signaling may be deleterious to at least a subset of cancer patients. For example, it has been shown in several types of cancer that progressive epigenetic silencing of TGFβ receptor I and/or II correlates with malignant progression. Pharmacological inhibition of TGFβ receptor signaling may promote metastases in these patients.

## Materials and Methods

All experiments involving animals were approved by the University of Nebraska Medical Center Institutional Animal Care and Use Committee (IACUC #: 07-047-08-FC).

### Cell Culture and Reagents

The FET [13] , GEO [8] and CBS [34] colon carcinoma cells are routinely maintained in a serum free (SF) medium containing insulin (20 ug/ml, Sigma) transferrin (4 ug/ml, Sigma) and EGF (5 ng/ml; R&D Systems) as previously described [46]. Cells were harvested after the addition of TGFβ (5 ng/ml) at the specified times. The TGFβRII, AKAP149, AKAP220 and PKA and PP2A catalytic subunit antibodies were obtained from Santa Cruz Biotechnology. The XIAP antibody was obtained from Abcam and actin from Sigma. The survivin, TGFβRI, AKT, Smad3, pSmad3, CREB, and IκBα antibodies as well as the PKA inhibitor H89 were obtained from Cell Signaling Technology. The AKAP inhibitor Ht31 and the phosphatase inhibitor Okadaic acid were obtained from Calbiochem.

### Western Blotting and Immunoprecipitation

Cells were lysed in a Tris-HCl based buffer containing 0.5% NP-40 and appropriate protease and phosphate inhibitors as described previously [13]. Protein concentration was determined by bicinhoninic acid assay (Pierce). Immunoprecipitation was performed with 500 ug protein aliquots using magnetic beads (Millipore) according to manufacturer's instructions.

### RNA Interference Studies

Smad 3 (sc-38376-SH), PKA Catalytic α (sc-36240-SH) and PP2A Catalytic α (sc-43509-SH) shRNA was obtained from Santa Cruz. These vectors were co-transfected by electroporation as described previously [34] with a puromycin selection vector and FET clones were selected in SF medium containing 4 ug/ml puromycin and 0.2% FBS. AKAP149 (sc-40301) and AKAP220 (sc-105049) siRNA and Transfection Reagent was obtained from Santa Cruz and knockdown was performed according to the manufacturer's protocol.

### PKA Activity Assay

PKA activity was measured using the PepTag non-radioactive protein kinase assay (Promega) using kemptide (LRRASLG) following the manufacturer's protocol. For quantitative determination of cellular cAMP, the non-radioactive Direct Cyclic AMP Enzyme Immunoassay kit (Assay Design) was used.

### cAMP Assay

For quantitative determination of cAMP, non-radioactive Direct Cyclic AMP Enzyme Immunoassay kit (Assay Design) was utilized. Manufacturer's protocol was followed for the assay.

### PP2A Assay

Protein Phosphatase 2A (PP2A) activity was measured using the non-radioactive PP2A immunoprecipitation assay (Millipore).

### Proteasomal Activity Assay

The fluorometric Proteasome substrate III for chymotrypsin (SUC-LLVY-AMC) and Peptidyl-glutamyl peptide hydrolyzing (PGPH)-caspase-like activity proteasome substrate II (Z-LLG-AMC) and the corresponding inhibitors were obtained from Calbiochem. FET cells were plated at 800,000 cells/100 mm dish in SF media. On day 5, cells were treated with TGFβ (5 ng/ml) with or without H89 (15 µM) pretreatment for 1 h and harvested in proteasomal assay buffer [50 mM HEPES (pH 7.5), 5 mM EDTA, 150 mM NaCl and 1% Triton X-100 containing 2 mM ATP]. After quantification of protein concentration, cell lysates were pretreated with 25 uM of the chymotrypsin-like activity inhibitor Benzyloxy carbonyl Leu Leu phenylalaninal Inhibitor (ZLLF-CHO) or the PGPH activity inhibitor Z-Gly-Pro-Phe-Leu-CH (Z-GPFL-CHO) followed by incubation with chymotrypsin or PGPH substrate for 1 h at 37°C as described by [47]. The chymotrypsin and PGPH activities were measured fluorometrically.

### Cell Death Assays

Apoptosis was measured by the Cell Death Detection ELISA Plus kit (Roche) as described previously [13]. Inhibition of cell proliferation was assessed by the MTT (3-(4,5-Dimethylthiazol-2-yl)-2,5-diphenyltetrazolium bromide) assay as described previously [48].

### Orthotopic Implantation for In Vivo Metastasis

All experiments involving animals were approved by the University of Nebraska Medical Center Institutional Animal Care and Use Committee. The orthotopic implantation methodology has been described in details in the following publications from Brattain laboratory [31], [32], [33]. Briefly, the GEO and GEORI cells used in orthotopic assays were transfected with Green Fluorescence Protein (GFP). Exponentially growing GFP-labeled cells were inoculated subcutaneously onto the dorsal surfaces of separate BALB/c nude male mice. Once xenografts were established (∼1 week), they were excised and minced into 1 mm^3^ pieces. These pieces were then orthotopically implanted into other BALB/c nude mice. For operative procedures, animals were anesthetized with isofluorane inhalation. A 1 cm laparotomy was performed and both the cecum and ascending colon were exteriorized. Using 7× magnification and microsurgical techniques, the serosa was disrupted by scraping in two locations. The 1 mm^3^ pieces of xenograft were sub-serosally implanted using an 8-0 nylon suture at the two points of serosal disruption. The bowel was then returned to the peritoneal cavity and the abdomen was closed with 5-0 vicryl suture. Subsequently, animals were anesthetized with a 1∶1 mixture of ketamine (10 mg/ml) and xylazine (1 mg/ml) by intraperitoneal injection (0.01 ml/mg) and weekly GFP fluorescence imaging was performed for up to 5 weeksWeekly GFP fluorescence imaging was performed for up to 5 weeks. Excitation of GFP in the light box facilitated identification of primary and metastatic disease by direct near-real time visualization of fluorescence in live animals. Both GEO and GEORI cells gave rise to an invasive primary tumor in 100% of the animals implanted.

### Hematoxylin and Eosin, TUNEL and Ki67 Staining

Approximately 50 d post-implantation, the animals were euthanized following proper IACUC protocol. The colon (with primary tumor), liver, lungs, and heart were harvested. Following the harvesting, organs were explanted, imaged, and immediately placed in 10% neutral buffered formalin fixative for 24 h. This was followed by tissues processing and embedding in paraffin. Slides were then cut for hematoxylin and eosin (H and E) and immunohistochemical characterizations. Serial sections were cut to complement the H and E sections and were stained with an IgG1 rabbit polyclonal antibody for Ki-67 (Dako North America, Inc., Carpinteria, CA). Slides from paraffin embedded tissue blocks were stained according to the Apotag (Oncor, Gaithersburg, MD) terminal nucleotidyl transferase-mediated nick end labeling (TUNEL) assay kit. Detailed methodology and quantitative analysis for these individual experiments has been described in details in the [32], [33], [49].

## Supporting Information

Figure S1 Activation of pSmad2.
(TIF)

Figure S2 PKA catalytic subunit shRNA knockdown in FET cells.
(TIF)

Figure S3 No change in IκB-α with TGFβ treatment.
(TIF)

Figure S4 Smad3 shRNA knockdown in FET cells.
(TIF)

Figure S5 TGFβ treatment downregulates survivin. Blocking the proteasome with MG132 abrogates the TGFβ mediated survivin loss.
(TIF)
