# Supplementary figures and images for: Identification of a Novel TGFβ/PKA Signaling Transduceome in Mediating Control of Cell Survival and Metastasis in Colon Cancer
Source: PLoS One. 2011 May 3;6(5):e19335. doi: 10.1371/journal.pone.0019335 (PMC3086924; doi:10.1371/journal.pone.0019335)

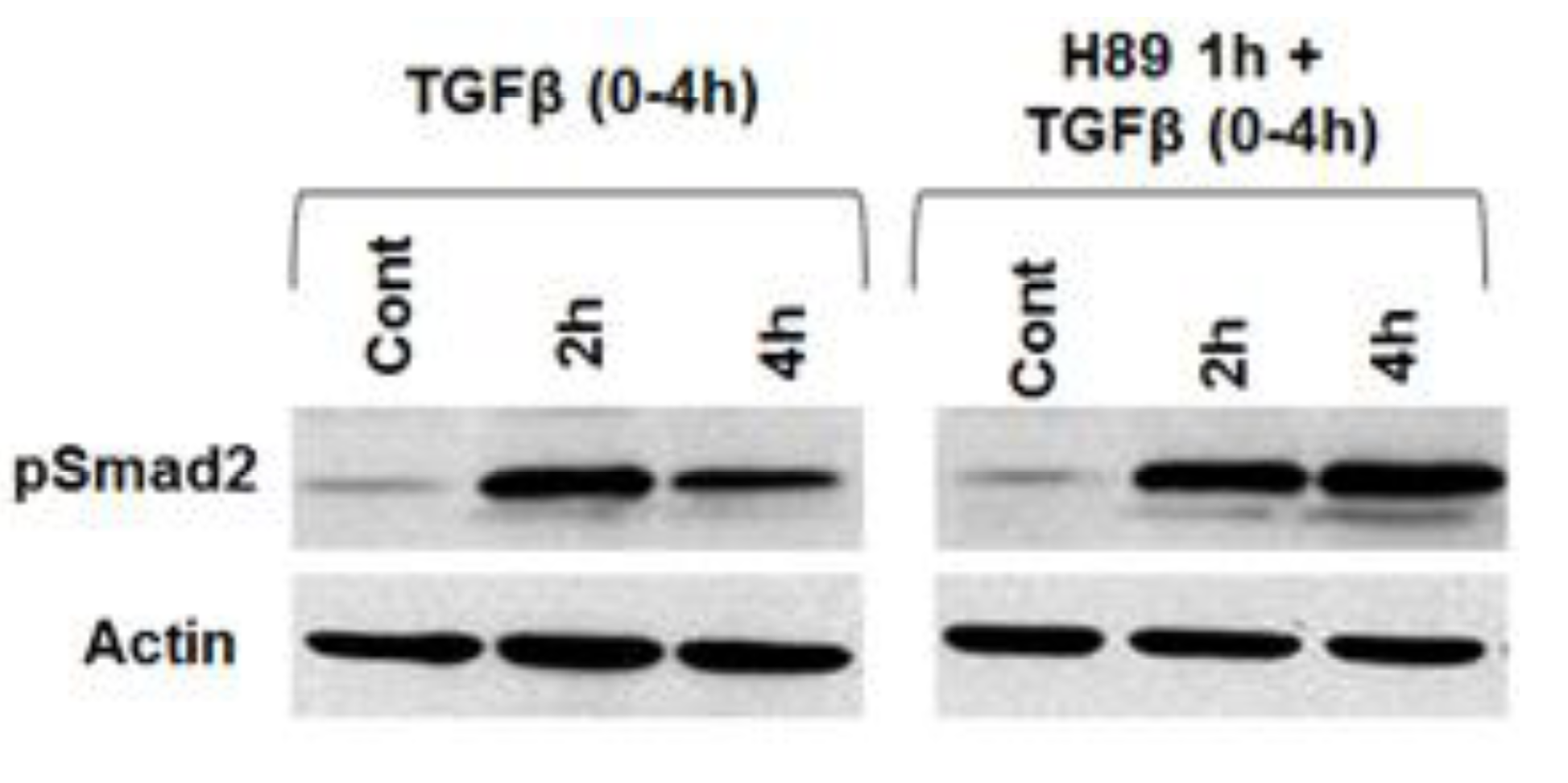

Supplement: Figure S1 — Activation of pSmad2. (TIF) [file pone.0019335.s001.tif]

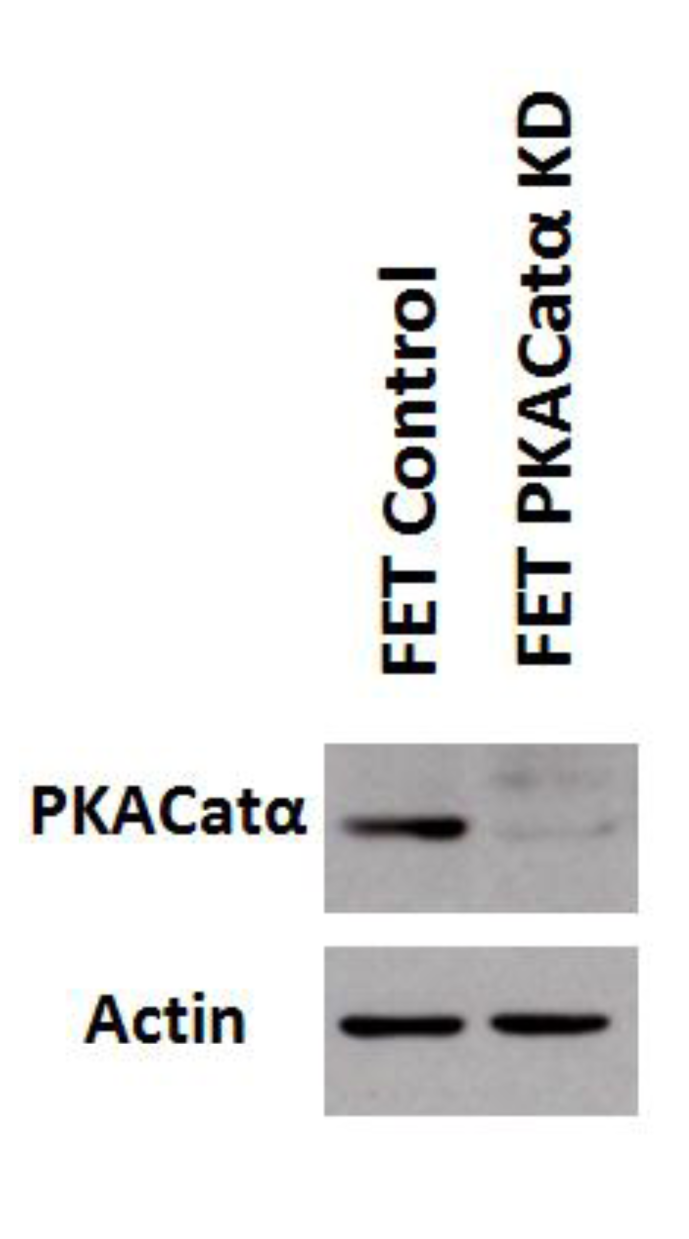

Supplement: Figure S2 — PKA catalytic subunit shRNA knockdown in FET cells. (TIF) [file pone.0019335.s002.tif]

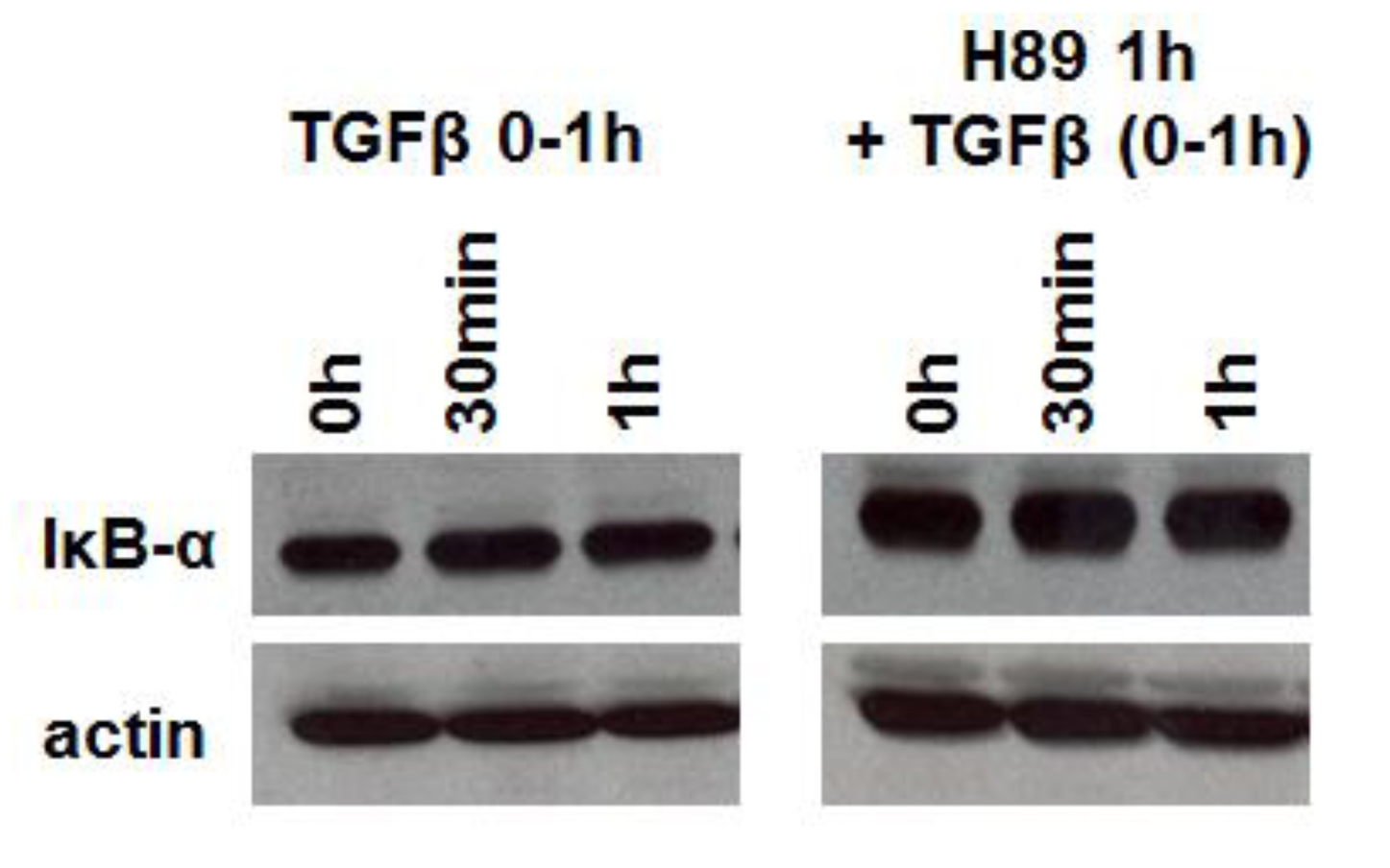

Supplement: Figure S3 — No change in IκB-α with TGFβ treatment. (TIF) [file pone.0019335.s003.tif]

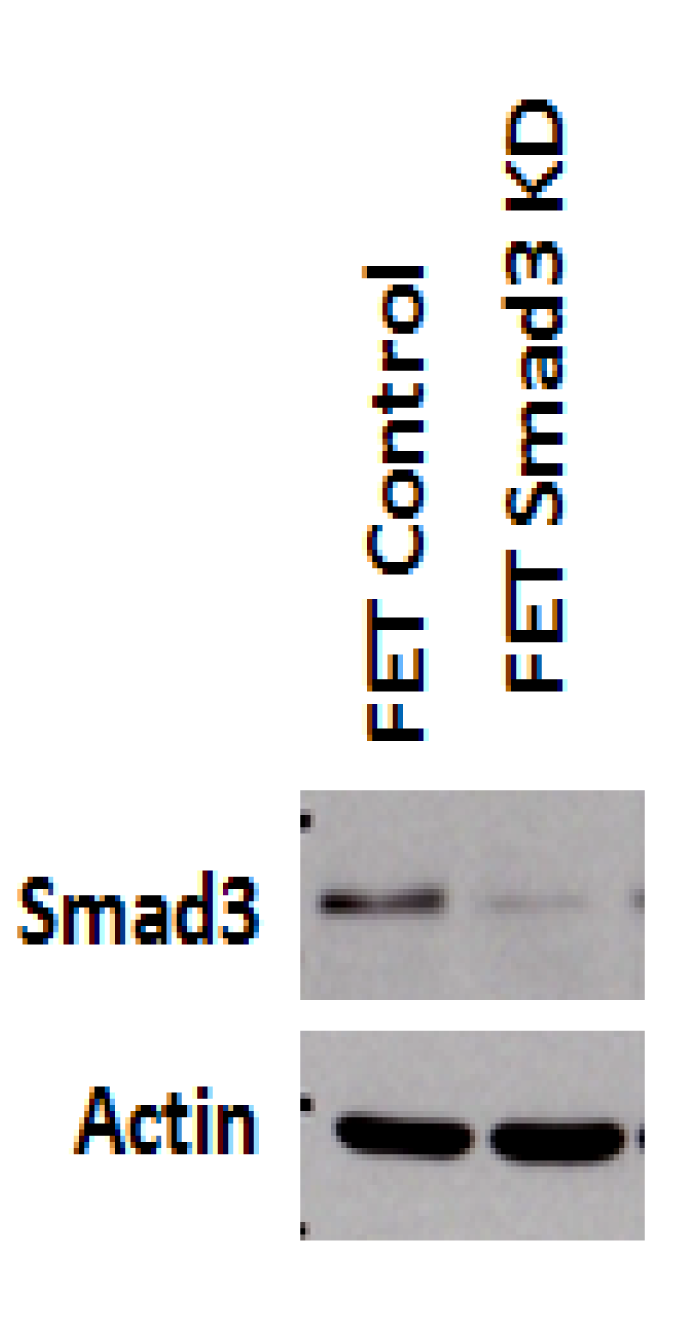

Supplement: Figure S4 — Smad3 shRNA knockdown in FET cells. (TIF) [file pone.0019335.s004.tif]

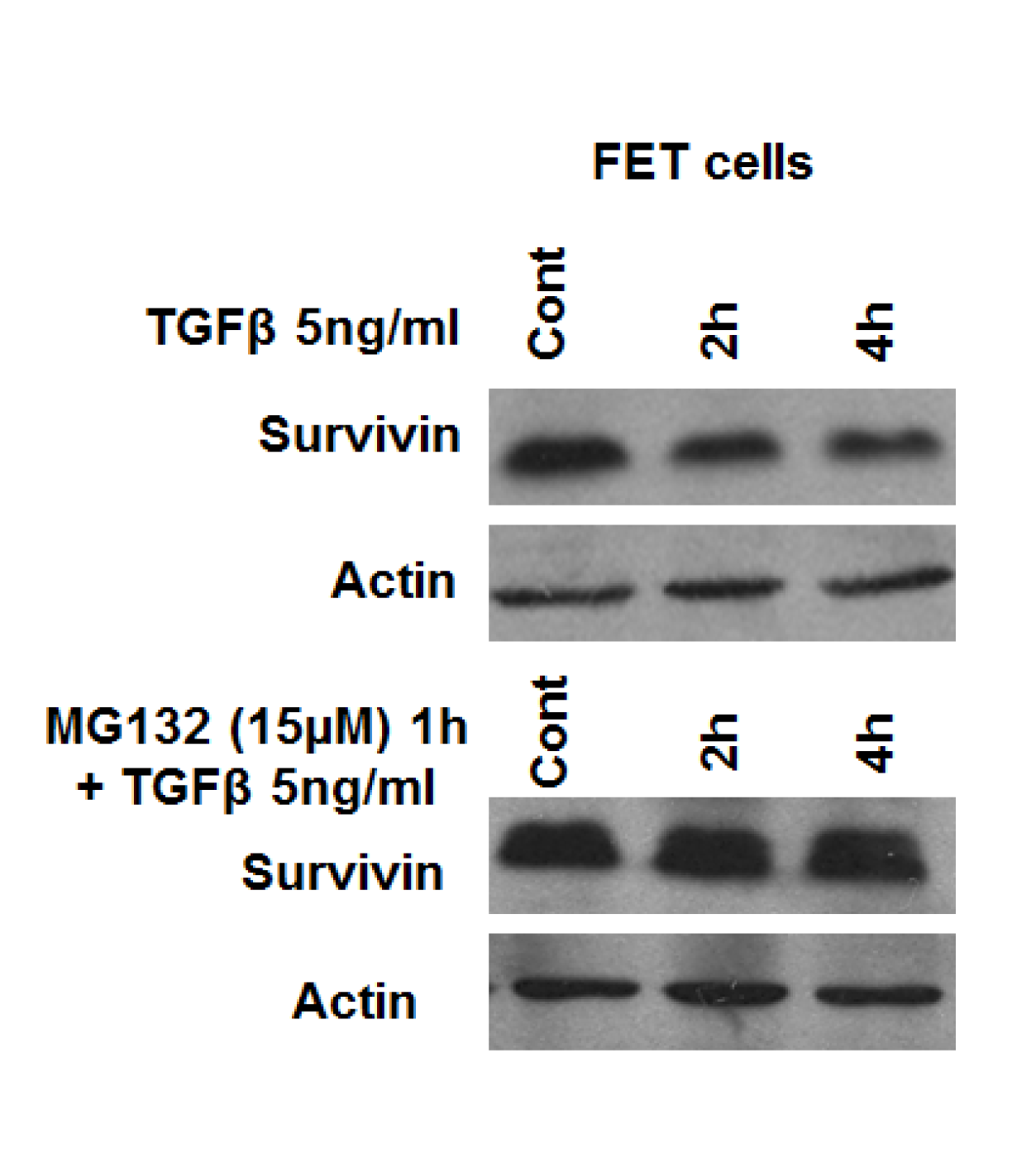

Supplement: Figure S5 — TGFβ treatment downregulates survivin. Blocking the proteasome with MG132 abrogates the TGFβ mediated survivin loss. (TIF) [file pone.0019335.s005.tif]
